# Identities between harmonic, hyperharmonic and Daehee numbers

**DOI:** 10.1186/s13660-018-1757-0

**Published:** 2018-07-11

**Authors:** Seog-Hoon Rim, Taekyun Kim, Sung-Soo Pyo

**Affiliations:** 1 Department of Mathematics Education, Kyungpook National University, Taegu, South Korea; 2 Department of Mathematics, Kwangwoon University, Seoul, South Korea; 3 Department of Mathematics Education, Silla University, Busan, Korea

**Keywords:** 05A19, 11B37, 34A30, Hyperharmonic numbers, Daehee numbers, Differential equation

## Abstract

In this paper, we present some identities relating the hyperharmonic, the Daehee and the derangement numbers, and we derive some nonlinear differential equations from the generating function of a hyperharmonic number. In addition, we use this differential equation to obtain some identities in which the hyperharmonic numbers and the Daehee numbers are involved.

## Introduction

For any *n*, we denote by $(x)_{n}$ the falling factorial $(x)_{0} =1, (x)_{n} = x(x-1)(x-2) \cdots (x-n+1)$ and $\langle x\rangle _{n}$ for rising factorial $\langle x\rangle _{0} =1, \langle x\rangle _{n}= x(x+1)(x+2) \cdots (x+n-1)$. Formally, $(x)_{n} = \langle x\rangle _{n} =0$ if $n < 0$.

The Stirling numbers are defined by $x^{n}$ and $(x)_{n}$ as

$$ \begin{aligned}& x^{n}= \sum_{k=0}^{n} S_{2} (n, k) (x)_{k}, \\ & (x)_{n}= \sum_{k=0}^{n} S_{1} (n, k) x^{k}, \end{aligned} $$

where $S_{1} (n,k)$ and $S_{2} (n,k)$ are called the Stirling numbers of the first kind and the second kind, respectively.

As is well known, the unsigned Stirling numbers of the first kind, denoted by $\vert S_{1}(n,k) \vert $, are $(-1)^{n+k}S_{1} (n,k)$. The unsigned Stirling numbers of the first kind $\vert S_{1}(n,k) \vert $ count the number of permutations of *n* elements with *k* disjoint cycles and the definition is given by

$$ \langle x\rangle _{n} = \sum _{k=0}^{n} \bigl\vert S_{1} (n, k) \bigr\vert x^{k}. $$

A derangement is a permutation of the elements of a set, such that no element appears in its original position. In other words, derangement is a permutation that has no fixed points. The number of derangements of a set of size *n*, denoted by $d_{n}$, is called the *nth derangement number*. The generating function of derangement numbers is given by

$$ \frac{e^{-t}}{1-t} = \sum_{n=0}^{\infty} d_{n} \frac{t^{n}}{n!}. $$

The *Cauchy numbers of order* *r*, denoted by $C_{n}^{(r)}$, are defined by the generating function to be

$$ \biggl( \frac{t}{\log(1+t)} \biggr)^{r} = \sum _{n=0}^{\infty} C_{n}^{(r)} \frac{t^{n}}{n!}. $$

It is well known that the *n*th *harmonic numbers*, denoted by $H_{n}$, are defined by

1 $$\begin{aligned} H_{n} = \frac{1}{1} + \frac{1}{2} + \frac{1}{3} + \cdots + \frac{1}{n} \end{aligned}$$

with $H_{0} = 0$.

The harmonic numbers have many applications in combinatorics and other areas. Several interesting properties of harmonic numbers can be found in [10].

In [3], the *nth hyperharmonic numbers* of order *r*, denoted by $H_{n}^{(r)}$, are defined by

2 $$\begin{aligned} H_{n}^{(r)} = \textstyle\begin{cases} 0 & \text{if } n \le 0 \text{ or } r< 0, \\ \frac{1}{n} & \text{if } n > 0 \text{ and } r = 0, \\ \sum_{i=1}^{n}H_{i}^{(r-1)} & \text{if } r,n \ge 1. \end{cases}\displaystyle \end{aligned}$$

From (1) and (2), we note that $H_{n}^{(1)} $ is the ordinary harmonic number $H_{n}$. Many authors have studied the hyperharmonic numbers [1–3, 5, 10, 21].

The Daehee numbers, denoted by $D_{n}$, are defined by the generating function to be

3 $$ \frac{\log(1+t)}{t}= \sum_{n=0}^{\infty} D_{n} \frac{t^{n}}{n!}. $$

It is clear that

4 $$ D_{0} = 1,\qquad D_{1} = - \frac{1}{2}, \ldots, D_{n} = (-1)^{n} \frac{n!}{n+1}. $$

The Daehee numbers serve as an intermediate medium connecting between several special numbers [6, 7, 11, 13, 31]. The higher-order Daehee numbers led to many combinatorial identities [8, 9, 13, 19, 22, 24, 29, 30]. In addition, the degenerate Daehee numbers have been defined and studied [13, 29, 30]. Recently many interesting results have been published regarding the degenerate Daehee numbers.

The *higher-order Daehee numbers*, denoted by $D_{n}^{(r)}$, are defined by the generating function,

5 $$ \biggl( \frac{\log(1+t)}{t} \biggr)^{r} = \sum _{n=0}^{\infty} D_{n}^{(r)} (x) \frac{t^{n}}{n!}\quad \text{(see [8, 9, 13, 19, 22, 24, 29, 30])}. $$

Recently, a group of mathematicians used a differential equations to study special numbers. In [11, 13], the Daehee and degenerate Daehee numbers are considered by using differential equations arising from the generating function. The identities for ordered Bell numbers [12] and Bernoulli numbers of the second kind [14] were derived arising from the differential equations of the generating functions, and the other identities of special polynomials can be found in [15–18, 20, 23, 25–27]. In this paper, we present some identities between the Daehee and hyperharmonic numbers. In addition, we derive some nonlinear differential equations from the generating function of the hyperharmonic number. In addition, we use this differential equations to obtain some identities in which the hyperharmonic numbers and the Daehee numbers are involved.

## Harmonic numbers and hyperharmonic numbers

Since $-\log(1-t) = t+ \frac{t^{2}}{2} + \frac{t^{3}}{3}+ \cdots$ , the generating function of the harmonic numbers $H_{n}$ is as follows:

6 $$ - \frac{ \log(1-t)}{1-t} = \sum_{n=0}^{\infty} H_{n} t^{n}. $$

From the definition of hyperharmonic numbers (2) and the generating function of harmonic numbers (6), we get the generating function of the hyperharmonic numbers:

7 $$ - \frac{ \log(1-t)}{(1-t)^{r}} = \sum_{n=0}^{\infty} H_{n}^{(r)} t^{n}. $$

The generating functions of harmonic and hyperharmonic numbers can be found in [1, 5] and [4].

A recurrence relation of the hyperharmonic numbers can be obtained by the generating function as follows:

$$\begin{aligned} &{-} \frac{ \log(1-t)}{(1-t)^{r}} (1-t) = \sum_{n=0}^{\infty} H_{n}^{(r)} t^{n} - \sum _{n=0}^{\infty} H_{n}^{(r)} t^{n+1} \\ &\phantom{{-} \frac{ \log(1-t)}{(1-t)^{r}} (1-t) }= \sum_{n=0}^{\infty} H_{n}^{(r)} t^{n} - \sum_{n=1}^{\infty} H_{n-1}^{(r)} t^{n} \\ &\phantom{{-} \frac{ \log(1-t)}{(1-t)^{r}} (1-t) }= \sum_{n=0}^{\infty} \bigl( H_{n}^{(r)} - H_{n-1}^{(r)} \bigr) t^{n}, \\ &{- }\frac{ \log(1-t)}{(1-t)^{r}} (1-t) = - \frac{ \log(1-t)}{(1-t)^{r-1}} \\ &\phantom{{- }\frac{ \log(1-t)}{(1-t)^{r}} (1-t) }= \sum_{n=0}^{\infty} H_{n}^{(r-1)} t^{n}. \end{aligned}$$

Therefore we get

8 $$ H_{n}^{(r)}= H_{n-1}^{(r)} + H_{n}^{(r-1)}\quad \text{for } n \ge 1. $$

This recurrence relation (8) is shown in [1], which we obtained in another way.

We note that, for $1 \le s \le r$,

9 $$\begin{aligned} - \frac{\log(1-t)}{(1-t)^{r}} &= - \frac{\log(1-t)}{(1-t)^{r-s}} \frac{1}{(1-t)^{s}} \\ &= \sum_{l=0}^{\infty} H_{l}^{(r-s)} t^{l} \sum_{k=0}^{\infty} \binom{-s}{k} (-1)^{k} t^{k} \\ &= \sum_{l=0}^{\infty} H_{l}^{(r-s)} t^{l} \sum_{k=0}^{\infty} \binom{s+k-1}{s-1} t^{k} \\ &= \sum_{n=0}^{\infty} \sum _{l=0}^{n} H_{l}^{(r-s)} \binom{s+n-l-1}{s-1} t^{n}. \end{aligned}$$

Equation (9) yields some identities that are presented in [1] as follows:

10 $$ \begin{aligned} H_{n}^{(r)} &= \sum _{m=1}^{n} \binom{n+r-m-1}{ r-1} \frac{1}{m}, \\ H_{n}^{(r)} &= \sum_{m=1}^{n} \binom{n+r-m-s-1}{ r-s-1} H_{m}^{(s)},\quad 0 \le s \le n-1. \end{aligned} $$

## Relations between hyperharmonic numbers and Daehee numbers

From the definition of Daehee numbers, we obtain

11 $$\begin{aligned} \frac{\log(1+t)}{t} &= \frac{- \log(1+t)}{(1+t)} \frac{1+t}{ -t} \\ &= \sum_{n=1}^{\infty} (-1)^{n+1} H_{n} t^{n} \biggl( 1 + \frac{1}{t} \biggr) \\ &= \sum_{n=1}^{\infty} (-1)^{n+1} H_{n} t^{n} + \sum_{n=0}^{\infty} (-1)^{n} H_{n+1} t^{n} \\ &= \sum_{n=1}^{\infty} \bigl( (-1)^{n+1} H_{n} + (-1)^{n} H_{n+1} \bigr) t^{n} + H_{1}. \end{aligned}$$

Since $H_{n+1} -H_{n} = \frac{1}{n+1}$, from (4) and (11), we get

$$ D_{0} = H_{1},\qquad D_{n} = (-1)^{n} n! ( H_{n+1} - H_{n} ) \quad\text{for } n \ge 1. $$

Let us investigate the relationship between the Daehee and hyperharmonic numbers.

12 $$\begin{aligned} \frac{\log(1+t)}{t} &= \frac{- \log(1+t)}{(1+t)^{r}} \frac{(1+t)^{r}}{ -t} \\ &= \sum_{i=1}^{\infty} (-1)^{i+1} H_{i}^{(r)} t^{i-1} \sum _{j=0}^{\infty} \binom{r}{j} t^{j} \\ &= \sum_{i=0}^{\infty} (-1)^{i} H_{i+1}^{(r)} t^{i} \sum _{j=0}^{\infty} \binom{r}{j} t^{j} \\ &= \sum_{n=0}^{\infty} \sum _{i=0}^{n} (-1)^{i} \binom{r}{n-i} H_{i+1}^{(r)} t^{n}. \end{aligned}$$

From (12) and the definition of the Daehee numbers (3), we get the following identity.

### Theorem 1

*For any non*-*negative integer* *n*,

$$ D_{n} = n! \sum_{i=0}^{n} (-1)^{i} \binom{r}{n-i} H_{i+1}^{(r)}. $$

From (4), we note that $\sum_{i=0}^{n-1} \frac{ (-1)^{i} D_{i}}{i!} = H_{n}$. Theorem 1 yields the following identity:

$$ H_{n} = \sum_{i=0}^{n-1} \sum_{k=0}^{i}(-1)^{k+i} H_{k+1}^{(r)} \binom{r}{i-k}, \quad\text{for } n \ge 1. $$

Let us consider higher-order Daehee numbers:

13 $$\begin{aligned} \biggl( \frac{\log(1+t)}{t} \biggr)^{r} &= - \frac{\log(1+t)}{(1+t)^{k}} \biggl( \frac{\log(1+t)}{t} \biggr)^{r-1} \frac{(1+t)^{k}}{-t} \\ &= \Biggl( \sum_{i=0}^{\infty} (-1)^{i} H_{i+1}^{(k)} t^{i} \Biggr) \Biggl( \sum_{j=0}^{\infty} {D_{j}^{(r-1)}} \frac{t^{j}}{j!} \Biggr) \Biggl( \sum_{l=0}^{\infty} (k)_{l} \frac{t^{l}}{l!} \Biggr) \\ &= \Biggl( \sum_{i=0}^{\infty} (-1)^{i} H_{i+1}^{(k)} t^{i} \Biggr) \Biggl( \sum_{m=0}^{\infty} \sum _{j=0}^{m} \binom{m}{j}{D_{j}^{(r-1)}} (k)_{m-j} \frac{t^{m}}{m!} \Biggr) \\ &= \sum_{n=0}^{\infty}\sum _{i=0}^{n} \sum_{j=0}^{n-i} (-1)^{i} \binom{n-i}{j} \frac { (k)_{n-i-j} D_{j}^{(r-1)} H_{i+1}^{(k)}}{(n-i)!} {t^{n}}. \end{aligned}$$

The following is found in Eq. (13) along with the definition of higher-order Daehee numbers.

### Theorem 2

*For any non*-*negative integer* *n* *and* $k \ge 1$,

$$ D_{n}^{(r)} = n! \sum _{i=0}^{n} \sum_{j=0}^{n-i} (-1)^{i} \binom{n-i}{j} \frac { (k)_{n-i-j} D_{j}^{(r-1)} H_{i+1}^{(k)}}{(n-i)!}. $$

Now, we want to express $H_{n}$ as a summation of $D_{k}$. We have

14 $$\begin{aligned} -\frac{\log(1-t)}{1-t} &= \sum _{n=0}^{\infty} H_{n} t^{n} \\ &= \frac{ \log(1-t)}{-t} \frac{t}{ 1-t} \\ &= \sum_{i=0}^{\infty} (-1)^{i} \frac{D_{i}}{i!} t^{i} \sum_{j=1}^{\infty} t^{j} \\ &= \sum_{n=1}^{\infty} \sum _{j=0}^{n-1} (-1)^{j} \frac{D_{j}}{j!} t^{n}. \end{aligned}$$

By comparing coefficients of the first line and fourth line in (14), we get an obvious identity:

$$ H_{n} = \sum_{j=0}^{n-1} (-1)^{j} \frac{D_{j}}{j!}. $$

Let us observe the definition of hyperharmonic and Daehee numbers. We have

15 $$\begin{aligned} - \frac{\log(1-t)}{(1-t)^{r}} &= \frac{\log(1-t)}{-t} \frac{t}{(1-t)^{r}} \\ &= \sum_{k=0}^{\infty}(-1)^{k} D_{k} \frac{t^{k}}{k!} \sum_{l=0}^{\infty} (-r)_{l} (-1)^{l} \frac{t^{l+1}}{l!} \\ &= \sum_{k=0}^{\infty} (-1)^{k} D_{k} \frac{t^{k}}{k!} \sum_{l=1}^{\infty} (-r)_{l-1} (-1)^{l-1} l \frac{t^{l}}{l!} \\ &= \sum_{n=1}^{\infty} \sum _{k=0}^{n-1} \binom{n}{k} (-1)^{n-1} D_{k} (-r)_{k-1} (n-k) \frac{t^{n}}{n!}. \end{aligned}$$

Equation (15) yields Theorem 3.

### Theorem 3

*For any positive integer* *n*,

16 $$ n! H_{n}^{(r)} = \sum _{k=0}^{n-1} \binom{n}{k} (-1)^{n-1}(n-k) (-r)_{k-1} D_{k}. $$

Theorem 3 shows that hyperharmonic numbers, $H_{n}^{(r)}$, can be expressed as a kind of sum of Daehee numbers. Naturally we can think of whether it is possible to express the Daehee number $D_{n}$ in terms of the hyperharmonic numbers $H_{n}^{(r)}$.

Let us observe Eq. (15) from a different point of view:

17 $$\begin{aligned} - \frac{\log(1-t)}{(1-t)^{r}} &= \biggl( \frac{\log(1-t)}{-t} \biggr)^{r} \biggl( \frac{-t}{ \log(1-t)} \biggr)^{r-1} \frac{t}{(1-t)^{r}} \\ &= \sum_{k=0}^{\infty}(-1)^{k} D_{k}^{(r)} \frac{t^{k}}{k!} \sum _{l=0}^{\infty} (-1)^{l} C_{l}^{(r-1)} \frac{t^{l}}{l!} \sum_{m=0}^{\infty} (-r)_{m} (-1)^{m} \frac{t^{m+1}}{m!} \\ &= \sum_{k=0}^{\infty}(-1)^{k} D_{k}^{(r)} \frac{t^{k}}{k!} \sum _{l=0}^{\infty} (-1)^{l} C_{l}^{(r-1)} \frac{t^{l}}{l!} \sum_{m=1}^{\infty} (-r)_{m-1} m (-1)^{m-1} \frac{t^{m}}{m!} \\ &= \sum_{k=0}^{\infty}(-1)^{k} D_{k}^{(r)} \frac{t^{k}}{k!} \sum _{n=1}^{\infty} \sum_{l=0}^{n-1} \binom{n}{l} (-1)^{n-1} C_{l}^{(r-1)} (-r)_{n-l-1} (n-l) \frac{t^{n}}{n!} \\ &= \sum_{n=1}^{\infty} \sum _{k=0}^{n-1} \sum_{l=0}^{n-1} \binom{n}{k} \binom{n}{l} (-1)^{k+1} D_{n-k}^{(r)} C_{l}^{(r-1)} (-r)_{n-l-1} (n-l) \frac{t^{n}}{n!}. \end{aligned}$$

### Theorem 4

*For any positive integer* *n*,

18 $$ n! H_{n}^{(r)} = \sum _{k=0}^{n-1} \sum_{l=0}^{n-1} \binom{n}{k} \binom{n}{l} (-1)^{k+1} D_{n-k}^{(r)} C_{l}^{(r-1)} (-r)_{n-l-1} (n-l). $$

By multiplying the generating function of the hyperharmonic numbers by $e^{-1}$, the following can be observed:

19 $$\begin{aligned} - \frac{\log(1-t)}{(1-t)^{r}} e^{-t} &= \sum _{l=0}^{\infty} H_{l}^{(r)} t^{l} \sum_{k=0}^{\infty} \frac{(-1)^{k}}{k!} t^{k} \\ &= \sum_{n=0}^{\infty} \sum _{l=0}^{n} H_{l}^{(r)} \frac{(-1)^{n-l}}{(n-l)!} t^{n}. \end{aligned}$$

From (19), we get the following identity:

20 $$\begin{aligned} - \frac{\log(1-t)}{(1-t)^{r}} e^{-t} &= - \frac{\log(1-t)}{(1-t)^{r-1}} \frac{ e^{-t}}{1-t} \\ &= \sum_{l=0}^{\infty} H_{l}^{(r-1)} t^{l} \sum_{k=0}^{\infty} \frac{d_{k}}{k!} t^{k} \\ &= \sum_{n=0}^{\infty} \sum _{l=0}^{n} H_{l}^{(r-1)} \frac{d_{n-l}}{(n-l)!} t^{n}, \end{aligned}$$

where $d_{k}$ denotes the *k*th derangement number. From (19) and (20), we get the following identity.

### Theorem 5

*For any positive integer* *n*,

21 $$ \sum_{l=0}^{n} H_{l}^{(r)} \frac{(-1)^{n-l}}{(n-l)!} = \sum _{l=0}^{n} H_{l}^{(r-1)} \frac{d_{n-l}}{(n-l)!}, $$

*where* $d_{k}$ *denotes the* *kth derangement number*.

## Some identities of hyperharmonic numbers and Daehee numbers arising from differential equations

From now on, throughout this article, we set

$$ \begin{aligned} G & = G(t) =- \log(1-t), \\ F & = F(t) = \log(1+t), \end{aligned} $$

and

$$ \begin{aligned}& F^{N} =\underbrace{F \times\cdots \times F}_{N\text{-times}}, \\ &F^{(0)}= F,\qquad F^{(N)} = \frac{d}{d t} F^{(N-1)}. \end{aligned} $$

In [28], Kwon et al. showed that $F= F(t) = \log(1+t)$ is a solution of the following differential equation:

22 $$ F^{(N)}= (-1)^{N-1} (N-1)! \sum _{n=0}^{\infty} (-1)^{n} N^{n} \frac{F^{n}}{n!}. $$

From Eq. (22), some relationships between the Daehee numbers and other special numbers have been found [28].

In [28], the authors presented two identities,

23 $$\begin{aligned} F^{(N)} &= (-1)^{N-1} (N-1)!\sum _{n=0}^{\infty} \Biggl( \sum _{m=0}^{n}(-1)^{m} N^{m} S_{1} (n,m) \Biggr) \frac{t^{n}}{n!} \\ &= \sum_{n=0}^{\infty} (n+N) D_{n+N-1} \frac{t^{n}}{n!}. \end{aligned}$$

From the definition of *G*, we get

24 $$ G' = \frac{1}{1-t} = e^{- \log(1-t)} = e^{G}. $$

By differentiation of both sides of Eq. (24), we get

$$ \begin{aligned}& G''= e^{G} G' = e^{G} e^{G} = e^{2G}, \\ &G^{(3)}= e^{2G} (2 G)' = 2 e^{3G}. \end{aligned} $$

By repeating this process, we can easily get

25 $$ G^{(N)} = (N-1)! e^{NG},\quad \text{for } N \ge 1. $$

From Eq. (12), we obtain

26 $$\begin{aligned} G^{(N)} &= (N-1)! e^{NG} \\ &= N! \sum_{m=0}^{\infty} N^{m-1} \frac{ G^{m}}{m!} \\ &= N! \sum_{m=0}^{\infty} N^{m-1} \frac{ (- \log(1-t) )^{m}}{m!} \\ &= N! \sum_{m=0}^{\infty} N^{m-1} (-1)^{m} \sum_{n=m}^{\infty} (-1)^{n} S_{1} (n,m) \frac{t^{n}}{n!} \\ &= N! \sum_{n=0}^{\infty} \sum _{m=0}^{n} N^{m-1} (-1)^{n+m} S_{1} (n,m) \frac{t^{n}}{n!}. \end{aligned}$$

From the definition of *G* and the hyperharmonic numbers,

27 $$\begin{aligned} G^{(N)} &= \biggl( \frac{d}{dt} \biggr)^{N} \biggl( \frac{-\log(1-t)}{(1-t)^{r}} \cdot (1-t)^{r} \biggr) \\ &= \biggl( \frac{d}{dt} \biggr)^{N} \Biggl( \sum _{m=0}^{\infty}H_{m}^{(r)} t^{m} \sum_{k=0}^{\infty} \binom{r}{k}(-1)^{k} t^{k} \Biggr) \\ &= \biggl( \frac{d}{dt} \biggr)^{N} \Biggl( \sum _{n=0}^{\infty} \sum_{k=0}^{n} \binom{r}{n-k} (-1)^{n-k} H_{k}^{(r)} t^{n} \Biggr) \\ &= \sum_{n=0}^{\infty} \sum _{k=0}^{n} \biggl( \frac{d}{dt} \biggr)^{N} \binom{r}{n-k} (-1)^{n-k} H_{k}^{(r)} t^{n} \\ &= \sum_{n=N}^{\infty} \sum _{k=0}^{n} \binom{r}{n-k} (-1)^{n-k} H_{k}^{(r)} (n)_{N} t^{n-N} \\ &= \sum_{n=0}^{\infty} \sum _{k=0}^{n+N} \binom{r}{n+N-k} (-1)^{n+N-k} H_{k}^{(r)} (n+N)_{N} t^{n}. \end{aligned}$$

Equations (26) and (27) yield the following theorem.

### Theorem 6

*For any positive integer* *N* *and non*-*negative integer* *n*,

$$ \frac{1}{n!} \sum_{k=0}^{n} N^{k-1} (-1)^{k} S_{1} (n,k) = \binom{n+N}{N} \sum_{k=0}^{n+N} \binom{r}{n+N-k} (-1)^{N-k} H_{k}^{(r)}. $$

We note that

28 $$ F(t) = - G (-t). $$

From (28), we have

29 $$ F^{(N)}(t) = (-1)^{N+1} G^{(N)} (-t). $$

Apply (29) to (27), then

30 $$\begin{aligned} (-1)^{N+1} G^{(N)} (-t) &= (-1)^{N+1} \sum_{n=0}^{\infty} \sum _{k=0}^{n+N} \binom{r}{n+N-k} (-1)^{N-k} H_{k}^{(r)} (n+N)_{N} t^{n} \\ &= \sum_{n=0}^{\infty} \sum _{k=0}^{n+N} \binom{r}{n+N-k} (-1)^{k+1} H_{k}^{(r)} (n+N)_{N} t^{n}. \end{aligned}$$

The definition of the Daehee numbers (3), (29) and (30) yields the following identity. This is a kind of inversion formula associated with Theorem 3.

### Theorem 7

*For any positive integer* *N*,

$$ D_{n+N-1} = (n+N-1)_{N-1} \sum_{k=0}^{n+N} \binom{r}{n+N-k} (-1)^{k+1} H_{k}^{(r)}. $$

From the definition of higher-order Daehee numbers (5),

31 $$\begin{aligned} G^{m} &= \bigl( - \log(1-t) \bigr)^{m} \\ &= \biggl( \frac{ \log(1-t)}{-t} \biggr)^{m} t^{m} \\ &= \sum_{l=0}^{\infty} (-1)^{l} D_{l}^{(m)} \frac{t^{l+m}}{l!}. \end{aligned}$$

Let us observe Eq. (27) in a different way:

32 $$\begin{aligned} G^{(N)} &= (N-1)! e^{NG} \\ &= (N-1)! \sum_{m=0}^{\infty} N^{m} \frac{ G^{m}}{m!} \\ &= (N-1)! \sum_{m=0}^{\infty} \frac{N^{m}}{m!} \sum_{k=0}^{\infty} (-1)^{k} D_{k}^{(m)} (k+m)_{m} \frac{t^{k+m}}{(k+m)!} \\ &= (N-1)! \sum_{n=0}^{\infty} \sum _{k=0}^{n}(-1)^{k} \binom{n}{k} N^{n-k} D_{k}^{(n-k)} \frac{t^{n}}{n!}. \end{aligned}$$

From (27) and (32), we have a relation between hyperharmonic and higher-order Daehee numbers.

### Theorem 8

*For any positive integer* *N*,

$$ \begin{aligned} \sum_{k=0}^{n+N} & \binom{r}{n+N-k} (-1)^{n+N-k} H_{k}^{(r)} (n+N)_{N} n! \\ & = (N-1)! \sum_{k=0}^{n}(-1)^{k} \binom{n}{k} N^{n-k} D_{k}^{(n-k)}. \end{aligned} $$

Substituting $1-e^{t}$ instead of *t* at (14) and (15), we have

33 $$ \begin{aligned} G^{(N)} \bigl( 1 - e^{t} \bigr) &= (N-1)! \sum_{n=0}^{\infty} (-1)^{n} N^{n} \frac{t^{n}}{n!} \end{aligned} $$

and

34 $$\begin{aligned} & G^{(N)} \bigl( 1 - e^{t} \bigr) \\ &\quad= \sum_{m=0}^{\infty} \sum _{k=0}^{m+N} \binom{r}{m+N-k} (-1)^{N-k} H_{k}^{(r)} (m+N)_{N} \bigl(e^{t}-1 \bigr)^{m} \\ &\quad= \sum_{m=0}^{\infty} \sum _{k=0}^{m+N} \sum_{n=m}^{\infty} \binom{r}{m+N-k} (-1)^{N-k} H_{k}^{(r)} (m+N)_{N} m! S_{2} (n,m) \frac{t^{n}}{n!} \\ &\quad= \sum_{n=0}^{\infty} \sum _{m=0}^{n} \sum_{k=0}^{m+N} \binom{r}{m+N-k} (-1)^{N-k} H_{k}^{(r)} (m+N)_{N} m! S_{2} (n,m) \frac{t^{n}}{n!}. \end{aligned}$$

From (33) and (34), we have the following theorem.

### Theorem 9

*For any positive integer* *N* *and non*-*negative integer* *n*,

$$ (-1)^{n} (N-1)! N^{n} = \sum_{m=0}^{n} \sum_{k=0}^{m+N} \binom{r}{m+N-k} (-1)^{N-k} H_{k}^{(r)} (m+N)_{N} m! S_{2} (n,m). $$

## Results and discussion

In this paper, we have studied the harmonic, the hyperharmonic, the Daehee and the higher-order Daehee numbers which are different from the previous research articles. In Sect. 2, we present some elementary identities between the harmonic and the hyperharmonic numbers. In Sect. 3, we study some relations and properties for the harmonic and the hyperharmonic numbers, the Daehee and the higher-order Daehee numbers. Additionally, the derangement numbers and the Cauchy numbers are also studied in Sect. 3. In Sect. 4, we study a nonlinear differential equation arising from the generating function of the harmonic numbers and we give some identities of harmonic and hyperharmonic numbers, the Daehee and higher-order Daehee numbers which are derived from this nonlinear differential equation.

## Conclusion

For a long time, research on the harmonic numbers was mainly focused on the study of inequalities. In this paper, we tried to study of the inequalities of the harmonic numbers by showing the relationship between harmonic numbers with other special numbers.
